# The neurosciences and the search for a unified psychology: the science and esthetics of a single framework

**DOI:** 10.3389/fpsyg.2015.01467

**Published:** 2015-10-07

**Authors:** Henderikus J. Stam

**Affiliations:** Department of Psychology, University of CalgaryCalgary, AB, Canada

**Keywords:** unification, psychology, methodology, neurosciences, science studies

## Abstract

The search for a so-called unified or integrated theory has long served as a goal for some psychologists, even if the search is often implicit. But if the established sciences do not have an explicitly unified set of theories, then why should psychology? After examining this question again I argue that psychology is in fact reasonably unified around its methods and its commitment to functional explanations, an *indeterminate functionalism*. The question of the place of the neurosciences in this framework is complex. On the one hand, the neuroscientific project will not likely renew and synthesize the disparate arms of psychology. On the other hand, their reformulation of what it means to be human will exert an influence in multiple ways. One way to capture that influence is to conceptualize the brain in terms of a technology that we interact with in a manner that we do not yet fully understand. In this way we maintain both a distance from neuro-reductionism and refrain from committing to an unfettered subjectivity.

## Will the Neurosciences Save Psychology or Can We Finally Give up the Search for a Single Framework?

In *The Shaking Woman* novelist and essayist Siri Hustvedt (2010, p. 3) described her experience of giving a talk in honor of her father some years after his death,

Confident and armed with index cards, I looked out at the fifty or so friends and colleagues of my father’s who had gathered around the memorial Norway spruce, launched into my first sentence, and began to shudder violently from the neck down. My arms flapped. My knees knocked. I shook as if I were having a seizure. Weirdly, my voice wasn’t affected. It didn’t change at all. Astounded by what was happening to me and terrified that I would fall over, I managed to keep my balance and continue, despite the fact that the cards in my hands were flying back and forth in front of me. When the speech ended, the shaking stopped.

Hustvedt (2010) describes her journey in coming to an understanding of this strange phenomenon, aptly captured by the subtitle of the book “a history of my nerves.” Moving through the worlds of neuroscience, psychoanalysis, psychology, psychiatry, and history, no one of them singly ever explains her strange experience. Of course the narrative is what matters in such accounts, the search through a contemporary knowledge of the brain and/or the mind, depending on one’s orientation, for a solution that no discipline by itself can easily muster. Instead, it becomes a story of how a self mysteriously aligned with a brain makes sense of unusual or difficult experiences. Such narratives are numerous; they populate not only the works of such well-known authors as Oliver Sacks, but constitute a genre by itself—the “brain memoirs” (Tougaw, 2012). Variants of illness narratives, these accounts are important for helping us understand the explosion of science as well as its limits. For if there were a straightforward neurobiological explanation there would be less of a detective story to tell and we would have more of a straightforward illness narrative instead.

What these narratives provide by way of a subplot is the failure of the mind/brain sciences to understand the complexities of the non-representative and non-standard case. But for authors, such as Hustvedt, who have immersed themselves in the philosophy and science of the brain, there is a keen awareness of the various sciences grappling with its subject matter. Such brain memoirs make fascinating reading not only for the overlapping questions of a self and its brain, but of the sciences of those selves and brains that are forever tripping over themselves to make the necessary connections between that self and that brain. And there are those who are now quite confident that the solution to all such mysteries lies in these brain sciences. Indeed, once remote and inaccessible, it now appears to certain authors, psychologists, and neuroscientists (Churchland, 2007) that the brain will soon integrate the various elements of psychology into a coherent science—finally a dream come true, one that has been articulated from the time of Julien de La Mettrie through to Karl Pribram and Antonio Damasio, and perhaps even Nikolas Rose. If we have seen this optimism before, it is likely because other projects were announced with equal optimism– behaviorism, cognitivism, and evolutionary psychology to name but a few. However, before I address these claims, I wish to ask instead what previous attempts at a grand synthesis have left us, and how much faith one ought to place in such attempts, for it is faith indeed that is required. Then I would like to dispel any entirely negative case by noting that certain kinds of synthetic frames can’t help but emerge from the neurosciences. The task is not to reject them but to understand and utilize those frames as appropriate tools.

## Modern Synthesis?

The possible options that qualify for a “modern synthesis^1^” seem more numerous today than even 30 years ago, when a version of evolutionary theory in its infancy, was one candidate. Psychology was largely dominated by a representationist and computational science of cognition. Do any of these, once hailed as revolutionary options, come close to providing us with a synthesis? This is a question that cannot be answered to any degree of satisfaction and we are far too early in the game to come to such a conclusion. It will be helpful, however, to consider other recent attempts to “unify” psychology. These will be useful for judging the adequacy of any particular attempt at “synthesis” be it neuroscientific or otherwise.

Let me make a simple claim at the outset. Historically sciences have ‘synthesized’, if at all, or become more or less integrated when they have found a problem or set of problems that promised resolution around some conceptual–methodological framework. Newton’s *Principia* is the token example and became a model for all subsequent attempts to resolve the question of just what a science ought to do to build a coherent framework. The many “Newtons” in natural philosophy who attempted to bring some rapprochement to the question of mind (think Kant and Hume, for example) only solidified this goal of a unified, mathematized vision of a science to which all should aspire. The messier biological sciences were never taken to be an aspirational model, although the modern synthesis in biology that united population biology with Mendelian genetics in the first half of the 20th century comes a close second (Mayr, 1942).

Nonetheless, it should be obvious that even after such unifications or syntheses the sciences in question did not fall into line. Physicists continued to argue, as did chemists and other practitioners of the new sciences in the 19th century. Hence, a synthesis was often a broad framework or set of problems that provided the necessary grounding for groups of scholars and scientists to proceed with the work of clearing the ground for further research. In the Kuhnian tradition, this came to be known as “normal science”, that is the science that carries on solving puzzles that might remain while some set of larger questions have been acceptably resolved (Kuhn, 1962/1996). So-called “revolutions” according to Kuhn (1962/1996) were major breakthroughs in the way in which science constituted its subject matter. Although Kuhn’s (1962/1996) version of this has in retrospect appeared overly simplistic and has been thoroughly debated, the question remains of just how major shifts in science occur and if they are at all predictable. For example, Mendeleev’s contributions to the table of elements not only appeared to “unify” chemistry but created the framework for discovering other elements that were not yet included in the table and would continue to be added to it—as they actually were as recently as only a few years ago. But not everyone is happy with the table of elements, for example earth scientists have found the traditional table to be quite limited in its applicability to geochemistry, mineralogy, aqueous chemistry, and related sciences. Hence these sciences have structured the table quite differently, often repeating elements and organizing these by charge (Railsback, 2003). In short, while working out of a “synthesis” such a synthesis is never totalizing, or complete. It shapes the established sciences by framing a broad consensus but any aspect of that consensus could break open at a moment’s notice under the right conditions.

It is obvious that the human sciences have never had such a stable framework. One might argue that the very idea of a human science, and a science of psychology in particular, was made possible by virtue of its ability to ignore much of what was relevant and important to human subjectivity by focusing, as Wundt originally had it, on the most simplified forms of human activity in the realm of perception and sensation. Already in the late 19th century the German debate about the relative importance of *verstehen* vs. *erklären* indicated a deep divide between what would inform, in part, a distinction between the human and natural sciences. Can one ever explain primary experience, consciousness and the like or are we, as participants in the phenomena we wish to explain always laboring on the margins of what is better understood from the first person perspective? When Snow (1959) turned his Rede lecture into an influential book (*The two cultures and the scientific revolution*), the divide between what we may call two fundamental forms knowing was considerable^2^. Psychology has continually attempted to straddle that divide, with occasional successes but largely with an inability to address the interpretive nature of human subjectivity at the expense of finely honed studies that address questions manageable or manufactured in the laboratory (Gergen, 1973; Robinson, 1985; Stam, 2012).

Every major development in the history of 20th century psychology has sold itself as a complete psychology. From behaviorism to evolutionary psychology through cognitive science and its multiple variants, the promulgation of a vast new theoretical framework was often accompanied by broad claims for its ability to be an absolute psychology. The failure of these projects was sometimes grand, as in the case of behaviorism, but often took the form of a disappointment that slowly led to the abandonment of whatever research models and projects were at hand. Business as usual in psychology was a “business” of determining just what constituted the phenomenon under investigation. This led to debates about intellectual territory, ideas, but most often, practices. For it was in the practice of psychology that the greatest advancement was to be found, the technologies of testing, behavior modification, therapy, counseling, personnel selection, and so on. These practices made enduring inroads into public acceptance and gradually managed to convince contemporary liberal democracies that psychology was an important if not always exact science that had much to offer in the form of technologies of classification, theories that focused on individuals as sources for problems of living, and general forms of practice the fit well with industrial and post-industrial societies. Hence while academic and research psychologists continued their long argument, applied psychologists got down to business and took care of their charges in clinics, schools, factories, offices, government, and elsewhere (Leary, 1987). It is not surprising then that contributions to theory were sometimes the outcome of broader changes in applied areas (e.g., the advances in statistical tools and interpretations derived from them that originated in applied fields of testing). This while psychologists in charge of the training of neophytes in the academies could not agree exactly on the nature of their science, nor on the precise mechanisms of intervention in the world.

This history is well known and I have merely given a brief summary here (Bühler, 1927; Vygotsky, 1927/1987; Koch, 1959–1963). But it has meant that from time to time there have been attempts to “unify” psychology under some banner or other so that, at the very least, the stories told to the public by both academics and practitioners would match. The claim is that psychology is not unified and this hurts both its practitioners and its status as a science (Staats, 1991; Henriques, 2008)^3^. A quick and simplistic comparison is then drawn with the natural sciences wherein physics is taken to be exemplary but even biology will do as a standard. This is then contrasted to psychology’s squabbles and the lack of a consensus on the status of just what is scientific and what counts as pseudo-science and, goes the argument, it is high time to clean up the mess. Some one or another scheme is then proffered for replacing many small but recalcitrant theories in the discipline and this over-riding scheme is usually packaged as superior because of its ability to unite, provide a foundation, or otherwise cohere the many strands that make up the contemporary discipline.

Although not numerous, such schemes usually include a list of reasons why this is a problem or why psychology is a “disunified science” in Staats’s (1991) words. After some broad generalizations, lumping all areas of psychology together, a wide variety of propositions or arguments have been put forth to unify the discipline. In Staats’s (1994) case, this was a “unified positivism” or a “psychological behaviorism” depending on what phase of Staats’s career one is reading. Ultimately it was an attempt to fuse multiple areas and features of psychology into a single “unified science.” Others of more recent vintage have attempted to keep these projects alive, or at least to put their personal stamp on such a project for every unification project seems to require that its proponent think through the problem anew. In recent years, Sternberg and Grigorenko (2001), Goertzen (2008) and Henriques (2008) among many others have continued to write on these questions, providing variations on the problem (is there a “crisis” of unification?) and offering numerous solutions (e.g., the “tree of knowledge,”—Henriques, a “unified psychology approach”—Sternberg), and so on (see Stam, 2004 for one critique).

The problems with these projects are (i) they are not responses to genuine problems in psychology but an attempt to impose order on disorder from an abstract vantage point, (ii) their relationship to empirical research is thin, and (iii) they rarely amount to more than a singular project or a personal vision of some abstract structures and/or institutional and political processes that might solve the so-called “crisis of disunification” (Green, 2015). But all of these, it is important to note, have also been proposed at a high level of abstraction without solving any particular, single, concrete problem in the discipline. Indeed what characterizes such projects is their considerable remove from the world of minute, everyday psychological phenomena.

To understand the way in which a modern synthesis might work it is important to understand first how it will *not* work. It is quite clear that all attempts at unification have been failures for multiple reasons. First, no serious science has ever been “unified” (assuming we actually know what this means), by a de facto decree. The history of science, however, is replete with examples, as noted above, of sciences that have coalesced around real problems that were genuinely altered by new methods, techniques, and theories that slowly—or quite suddenly—opened up new ways of examining traditionally recalcitrant problems. The closest to the sciences of the mind that might be relevant for all future investigations of syntheses is biology. It found its professional voice in the 19th century following the gradual acceptance of Darwin’s theory of natural selection and then found new momentum in the 20th with, as noted, the modern synthesis, or the combined forces of population biology and genetics which supported a broad understanding of evolution (Mayr, 1942). Nonetheless, in biology unification is still far off even if this has not prevented certain strategies of integration (Mitchell and Dietrich, 2006). This distinction between unification and integration is useful for the integration is based on actual empirical problems and examples, whereas unification is often an abstract proposal imposed from above. As Mitchell and Dietrich (2006, p. S78) note,

There are multiple mechanical triggers for behavior for a complex system. Which ones are present and active may well be a function of the ecological context in which the system is located. Explaining complex, evolved biological systems is not a “one-size- fits-all” enterprise.

In comparison, psychology has more or less shifted from one project to another, never entirely abandoning what went before but attempting each time to begin again on a new footing. Behaviorism incorporated elements of functionalism just as the new cognitive psychology adopted elements of Hullian behaviorism. These were always partial appropriations, and rhetorically behaviorism differentiated itself from functionalism just as cognitive psychology differentiated itself from behaviorism. Nevertheless, these breaks were never quite as clear as they appeared on the surface. However, more to the point, as Koch (1971, pp. 690–691) noted, “as for the *subject* matter of psychology, it is difficult to see how it could ever have been thought to be a coherent one under any definition of the presumptive ‘science,’ whether in terms of mind, consciousness, experience, behavior, or, indeed, molecule aggregates or transistor circuits”. Forgotten in all of this too is that there is no longer any center to the discipline of psychology, if there in fact ever was. To quote Koch (1971, p. 695) again, who proposed,

that the essential non-cohesiveness of the activities denoted by the term “psychology” be acknowledged by replacing it with some locution as “the psychological studies.” Students should no longer be tricked by a terminological rhetoric into the belief that they are studying a single discipline or any set of specialties rendered coherent by any actual or potential principle of coherence.The current “departments of psychology” should be called “departments of psychological studies”.

## Unified After All?

Despite this seeming disarray and ‘disunity’ of the discipline called psychology, there are in fact features that artificially but successfully have held the discipline together for more than half a century. For despite all the calls of crisis, psychology has been hugely successful if one only counts the number of psychologists plying their trade in such diverse domains as the classroom, the clinic, the workplace, and a multitude of laboratories around the world. As sociologists of the professions note, to be a successful discipline requires first, that one have a marketplace within which one can disseminate symbolic capital, second, an acceptable manner of producing knowledge, and third, a system of training to reproduce members of the discipline (Freidson, 1986). Psychology has had all three in abundance, and hence continues to thrive. But it is not enough to produce a stable discipline for, after all, phrenology also had all three but is no longer in evidence despite its immense popularity in the 19th century. We must look further then for the roots of this stability.

The other deeply rooted features of psychology that are easily reproduced even in such cases where no two psychologists agree on a fundamental framework are (i) psychology’s methods and (ii) its functional interpretation of just about any and all of its conceptual elements (Stam, 2004). The first is obvious, the second is much more subtle.

First, methods have become remarkably stable in the face of continuing disagreements and debates about the subject matter of psychology. It is as if, by tacit agreement, psychologists have come to realize that methodology is what holds their discipline together in the absence of any agreed upon frames of reference, common vocabulary or shared theoretical understandings (sometimes referred to, at least since Gordon Allport, as *methodolatry*). These methods include not only the common variety of methods taught in our universities, such as those associated with experimentation and quasi-experimentation but also include the statistical tools that are symbiotic with these methods, such as the analysis of variance in all its forms, regression models in its linear and non-linear forms, and also the multiple ways of producing items for such tools as psychometric instruments. More recently it has come to include, slowly but surely, the new forms of qualitative analyses and research, such as discourse analysis, grounded theory and so on. That this constitutes a common vocabulary of sorts for much of psychology is readily appreciable when one considers that the one feature psychologists from diverse fields hold in common is their common educational history in methods classes. They may not understand what their colleagues are up to but they can still critique their faulty use of a regression analysis!

Second, the general use of functional accounts (what I have called *indeterminate functionalism*; Stam, 2006, 2015), which have impregnated almost all forms of psychological work and theorizing, has rarely been the subject of much discussion.

Without them, however, it is hard to imagine how psychology would continue to reproduce itself. This is much less obvious nor as readily acknowledged among the halls of academe or in the clinics or workshops of the psychologist. A functional vocabulary refers to the notion that we are primarily interested in the functional properties of whatever it is we are investigating, treating, predicting, or otherwise describing. This is as true for behavioral, neuropsychological, cognitive, developmental, school, social, and whatever other areas of psychology that make up the contemporary discipline (it is not, however, universally true, there are exceptions). The point is that when we describe, say, a memory as a research object we do not have a material object in mind. We mean by a memory a kind of activity that is specified in research or practice as a recallable item of some sort that was either learned as part of an experiment or that involves some restricted or constrained recall of personal knowledge or events. But there are no objects called “explicit memory,” “short-term memory,” “procedural memory,” and so on in the way that we have, for example, mitochondria, or aminoglycosides or even something as complex as particles in linear accelerators that are only hypothesized to exist^4^. Our functional vocabulary identifies a psychological object by virtue of its existence as a function of some set of activities. This is as true for broad categories of psychological objects as for more delimited ones such as episodic memory. Think of extraversion, which is a standard component of many personality scales and has been for at least 60 years. We determine that someone is extraverted not from their conversational skills or their unwillingness to stand up and speak in public, but from their avowal or disavowal of items on a standardized personality inventory such as “I talk to a lot of different people at parties^5^.” In the act of agreeing with such items we come to recognize the object “extraversion” and assign it to individuals as an aggregate score whose value lies in its status as a normative score on some dimension, that is, a score that then allows us to compare this person to others who have responded to the same questions.

Thus far this is rather mundane if not obvious to students of psychology. How could it be otherwise one might ask? Well, various attempts have been made to provide more certain foundations for the choice of psychological objects, either in the form of a serious materialist reductive program, a radical behaviorist program or certain versions of cognitive psychology. Wouldn’t it be more stable if all psychologists ceased relying on any “verbal report” and instead chose to rely strictly on behavioral indices. Unfortunately the history of behaviorism has shown us that “behavior” is equally interpreted. When does a movement constitute behavior? How do we distinguish aggressive behavior from nurturing behavior except through a series of interpretive functional accounts we create in research studies. Suffice it to say that the vast majority of psychological theorizing takes place in the form of a functionalist framework, carrying on a long tradition that has its origins in 19th century physiology. On that score Wundt was certainly original insofar as he was able to bend the vagaries of certain psychological properties to his will by subjecting them to an experimental investigation and a functional account.

There is an obvious benefit to the way in which functional accounts are structured: the inherent flexibility of such accounts makes it possible to rapidly expand one’s theoretical armamentarium. For example, there are several hundred different kinds of memory (Tulving, 2007), dozens of types of personality scales with different numbers of not items but factors, innumerable variables under investigation in social psychology such that researchers have specialized in a few in their limited domain since no one can possibly grasp the whole, and so on. I can do a study on psychological factor *x* and decide legitimately that *x* is really not one but two factors, so I create *x*′ and *y*′. Someone else continues in this research and adds another variable to this configuration that is presumably responsible for both *x*′ and *y*′, and calls it *z*″. And before long we have not just a difference between episodic and semantic memory but also a distinction between declarative and procedural memory, explicit and implicit memory, short-term and long-term memory, and so on. Not that memory researchers start out with a single system and branch out, but that given any kind of memory, it is not difficult to refine and distinguish another memory based on variations in procedures used to elicit the memory.

It should be obvious that the inherent flexibility in identifying new functions that can be created in a research settings and then named as part of some functional account is not just important for its flexibility but is a process that can be carried on indefinitely.

There is no in principle limit to the kinds and number of functional ascriptions possible. Note that this is not a statement about the limits of science. There is also no in principle limit to the kinds and number of elements in the periodic table of elements. However, there is both a theoretical limit and an empirical constraint on just how large such a table can be despite the many additions to the table since Mendeleev’s time. In psychology, the empirical constraints are missing, one can always devise a new procedure in one’s research that will bring the new function into existence. One can devise, for example, a new memory task that will allow for the demonstration of a new form of remembering.

And the procedure of expanding the kinds of memories that exist would simply move forward. In that case we cannot speak of ‘empirical adequacy’ as it is sometimes used to describe a key characteristic of science. For the empirical procedure that calls the function into existence (e.g., episodic memory for events and experiences) is the same as the criterion of empirical adequacy, which demonstrates the fact of episodic memory. We are caught in a vicious circle since we have no ontological *a priori*.

It is generally assumed that functional accounts keep from slipping into dualism by virtue of their appeal to a series of promissory notes whose claim is that, eventually, a truly reductive account will reveal all. And memory researchers have, of course, provided numerous neurological candidates for various memory models (Eichenbaum and Cohen, 2004; Aggleton and Brown, 2006; Cohen, 2015). Hence, indeterminate functionalism could be made more determinate by fixing certain categories to neurological structures. This is after all one aim of cognitive neuroscience. It should be noted, however, that even when ‘fixed’ in this manner functional categories remain ambivalently indeterminate by virtue of the fact that they exist as procedures, not as objects.

In short, between our methods and our functional vocabularies and explanatory strategies, psychology is much more unified than seems the case on the surface. But it is also relatively incoherent; since that is what I think is often meant by “disunified.” The incoherence is the direct outcome of a lack of agreement on just what psychological objects are and how we might define them. Our functional strategies allow us to define new variables ad infinitum. Psychology appears to be all epistemology without a clear ontology.

## Applied Psychology: Applications of a Science?

Although this may seem a different question altogether, one driver of the flexible research programs that psychology promulgates has been the rather lopsided relationship between researchers and practitioners. The vast majority of psychologists currently active in the world work as practitioners. This means that they could be anything from clinical psychologists, counseling psychologists, educational psychologists, personnel psychologists, military psychologists, industrial psychologists, or a host of other applied professional psychologists working in varied settings. Numbers here are dubious, given that no one body is responsible for, or concerns itself with, tracking exact global numbers for professions. However, given that at least a quarter of psychologists in the world live and work in the US (about 160,200 by last count, Bureau of Labor Statistics, 2014), the vast majority (up to 90%) is estimated to be engaged in clinical, counseling or school activities or in health service provider and industrial–organizational activities.

These numbers generally reflect trends in the North Atlantic regions and demonstrate that psychologists who dominate the discipline are little concerned about the arcane features of academic debates that interest those in universities and research-only settings. It should be clear that practicing psychologists receive their education in universities but are generally not beholden to such principles as promised by a “scientist practitioner” model or the more recent minority view, the “clinical scientist” model. The question then is, can there ever be a genuine intellectual revolution that will provide a kind of synthesis for this wide range of activities. Koch thought it was an impossible task since there was no single discipline to unify. I wish to enquire what the neurosciences might offer.

## The Neuroscientific Synthesis

The spectacular advances in imaging techniques made possible by not only the refinement of electroencephalography (EEG) measures but by the addition of positron emission tomography (PET), computerized tomography (CT), optical tomography and functional magnetic resonance imaging (FMRI) scans has greatly advanced the “visibility” of brain processes even though each of these techniques are dependent on sophisticated statistical and constructive mathematical and computerized processes. These have gone with equally swift advances in research in the neurosciences, but as numerous “neuroskeptics” have pointed out (eg., Boekel et al., 2015), the science is hardly optimal and replications often fail. We are a long way from understanding just what the brain does and how it does it, but there is a general optimism that the neurosciences will save psychology and psychiatry from the repeated adoption of fad-like theories that are typically discarded after one or two generations (Bickle, 2003; Caruso, 2012; Reardon, 2014; but see Machamer and Sytsma, 2007). That optimism notwithstanding, the neurosciences indeed are a formidable interdisciplinary, multipronged and richly funded matrix of research, tools, and practices whose imagery creates at least the appearance of a science slowly but surely removing the veils of ignorance that have kept us from understanding ourselves.

And as Wittgenstein noted in a different context, it is just such an image that can hold us captive.

The question here is to what degree can the neuroscientific project renew and synthesize the disparate arms of psychology? Although popular books and articles appear at a steady rate, we are far too early in the game to provide any kind of answer to this question. What the neurosciences are unlikely to do is mimic their colleagues in evolutionary psychology, which has gone through a rather marked decline in the past decade. Following the revival of sociobiology under the guise of a modular evolutionary psychology (Tooby and Cosmides, 2005), it promised to be the new model for a revived integrated psychology. That this has not happened is due to many features of this new approach, not least of which is the rerun of similar issues that bedeviled sociobiology. Mostly, however, evolutionary psychologists relied heavily on the language of genetics to provide the justification for their hypotheses. Genuine genetic analyses were remarkably absent, however, from the work of evolutionary psychology (Dagg, 2005) and the recent science of epigenetics has made problematic much of evolutionary psychology’s claims [for a definition of epigenetics see Berger et al. (2009)]. As is the case for most theories, adjustments can and will be made to save the theory, however, it’s simplicity and purported broad applicability will suffer as a consequence.

Critics have worried that the neurosciences are either reductionist in their intent, with all of the problems that follow from this (Choudhury and Slaby, 2012), or they are subject to the mereological fallacy in which powers and activities are attributed to brains or parts of brains when these are normally ascribed to persons as a whole (cf. Bennett and Hacker, 2003; Gergen, 2010). Such critiques have their place, for surely much neuroscience is reductionist in intent. And the reductive language cannot help but fail to replace a language of meaning and intent. That is, a reductionist neuroscientific language cannot replace the reporting role of ordinary language, the language of intentions, semantics, and sentience. If it could, it would have to be as contextually sensitive as ordinary language and we would be back to where we began. However, reductive strategies for certain purposes are not only useful, as for example, in locating and treating disorders that may have their origin in the brain, but also for understanding the structure, function and neuropharmacological properties of brains.

Other critics have noted the limitations of neural processes in explaining complex social activities. For example, Coey et al. (2012) have noted that understanding the context of social interactions requires understanding their “embodied-embedded” constraints. These authors argue that the organization of human behavior, particularly its self-organizing processes, requires something much more dynamic than a neural account.

Given these limitations there will always be doubts about the overall “synthesizing” potential of the neurosciences. It would be a mistake, however, to dismiss the impact of the neurosciences on psychology and the shift that it will force on the discipline in the coming years. I take here as telling that Nikolas Rose is ambivalent since he is generally a critic of psychology and all “psy” disciplines, that is, those disciplines that are engaged in processes that he argues are invoked in practices of “governmentality” after Foucault, that refer in particular to the creation of subjectivities through the organized practices of a society. But, according to Rose and Abi-Rached (2013, p. 21), “despite their apparent contradictions, neurobiological research emphasizing the role of non-conscious neural processes and habits in our decisions and actions can-and does—happily coexist with longstanding ideas about choice, responsibility, and consciousness that are so crucial to contemporary advanced liberal societies.” That is, neuroscience has not removed from us our responsibility to be actors whose fates are not captured only by processes that occur outside awareness in our brains, but also has not lessened the requirement that we govern those forces through an endless process of self-discipline. Despite the fact that the neurosciences constitute “psy” disciplinarity by other means, it should be obvious argue Rose and Abi-Rached (2013, p. 21) that “human brains are both shaped by, and shape, their sociality”. What this leads to is a discourse (my term) of neuroscience that will ultimately move beyond a neuro-reductive language to one that will address “questions of complexity and emergence, and to locate neural processes firmly in the dimensions of time, development, and transactions within a milieu” (Rose and Abi-Rached, 2013, p. 23). In other words, the picture of our brain as plastic and ultimately social is a revisionist one that can be used for multiple ends.

In a related vein both Moore (2006) and Derksen (2011) have urged an alternative view of brains and evolution. Moore argued that it is more productive to think of evolutionary psychology as the outcome of the design and production of technical systems rather than engineered mechanisms. Its originality lies in its amalgamation of so-called standard adaptationist accounts of evolution with those that are interactionist and typically critical of adaptationist accounts. It hinges on a conception of technology as a set of social relations, leading to an evolutionary psychology that can account for the emergence of mindedness and sociality (Moore, 2006). Taking the argument of biology as technology seriously, Derksen (2011, p. 844) notes along with Andy Clark that “A technological conception of the brain leads away from neuro-reductionism rather than toward it, as long as one keeps an eye on the relational nature of the mechanisms that make up the mind, and one is willing to see the extension of the mind beyond the ‘skinbag’ into a growing network of tools” . The brain-as-instrument is an unusual reconceptualization argues Derksen (2011) because we are both identified with our brains and treat it as something external to us. The brain as instrument is an attempt to steer between a version of personhood that makes us neither the passive bystanders of what happens in “our brain” nor does it make us able to ‘use’ the brain just as we will.

Perhaps a return to one of Latour’s (2004) formulations might help here. Using the example of developing a “nose” for perfume he argues that what matters in learning to differentiate among many odors is the ability to *articulate* different odors after lengthy practice. It is not a question, for Latour, of determining the exact, precise chemical foundation of an odor, that is, to develop an *accuracy of reference*. As Latour (2004, pp. 210–211) argues, “the decisive advantage of articulation over accuracy of reference is that there is no end to articulation whereas there is an end to accuracy.”

Transposed to the brain sciences, what a technological conception provides us with is an ever greater possibility of articulation of just what the brain is capable of, how it makes a difference in life, what it allows us to do, and so on, without having to immediately decide that one is being neuro-reductionist or that one must defend against such a stance. Instead, brains, like eyes, ears, and noses, make articulations possible in ways we have not fully realized. Again, in the words of Latour (2004, p. 226), “It is not a fight against reductionism nor a plea for the whole personal, subjective body that should be respected instead of being ‘cut into pieces’.” Reductionism is on his account, simply an impossibility, just as having no body is an impossibility. So rather than creating a sharp division between reductionist science on the one hand and a militant subjectivity on the other, the question of the body (and the brain) is one of articulating the multiple possibilities and positions that emerge from the new sciences, not to determine where the objective body ends and the subjective body begins (see the program for a neurophenomenology as one attempt to develop research methods appropriate to a slightly alternative strategy, e.g., Olivares et al., 2015).

What this position attempts to do is to escape from the Scylla of reductionism and the Charybdis of subjectivity. Must we, with Metzinger (2009) who, in echoing Julien Offray de La Mettrie’s *L’homme machine*, proclaims that there is no self argue that we can never solve the problem of consciousness? Or must we privilege a stubborn subjectivity? What a technology or, perhaps better said, a techno-science position claims for the brain is nothing less than all there is to know about the brain. But all there is to know is not the end of the story, for what we come to know elides in multiple ways with the social world and is taken up as a problem for subjectivity. As a consequence we articulate, in Latour’s sense, the world differently. Just as people articulated the world differently after discovering that a heart was better thought of as a sophisticated pump. Or when it was discovered that electromagnetic radiation of very short wavelengths could penetrate matter to become what we now refer to as X-rays, this knowledge and everything it has revealed to us about the human body has been integrated into our practical knowledge of ourselves. When an X-ray of our broken wrist is displayed, we understand that this too is a part of us—both as object and as problem. As a technology it is both distancing and revealing. It looks like something other than us, while we recognize that it also reveals who we are and is made possible by a vast network of medical practice that has shaped bodily existence in the 20th century and beyond.

The brain-as-technology question is compounded, however, if not confounded, by reflexivity. Brains are not only technology, they are us and at the same time they are not us (Dotov et al., 2010). Hence how the brain sciences become integrated into contemporary medical, psychological (‘psy’ disciplines), and social disciplines and practices will reveal and depend on the interests of multiple actors and interests. What they won’t do is become the unifying theoretical edifice that psychologists have dreamt of for so long. However, they can open up not only new avenues for inquiry but also reveal new ways of being human. These new ways will not just supplant our older forms of self-understanding but will likely become integrated into what we already know ourselves to be. Just as psychoanalysis did not destroy the western conception of personhood, it did open up alternative questions, modes of thinking, and moral frameworks that had not been obvious or present before psychoanalysis. Once psychoanalysis had become deeply embedded in contemporary culture there was no way back to a late-19th century view of mind and human nature, at least not for the citizen of the modern, that is, post-WWI western world. Psychoanalysis grew up with and has become ensnared in industrialized societies and as these societies shifted to broadly post-industrial, globalized forms of neo-liberalism the explanatory forms of psychoanalysis were unable to sustain the versions of personhood emerging. As the neurosciences feed into our contemporary versions of fragmented personhood, they too will elaborate, differentiate and contribute to renewed models of persons. Indeed, even psychoanalysis has become neuropsychoanalysis (Solms and Turnbull, 2011).

Perhaps the law can serve as an illustration. Neuroscience, like any science potentially, can affect legal cases wherever that science is relevant. But neuroscience has a unique role in so far as it will lead the legal system to question key notions of responsibility that are central to determinations of guilt or innocence. As Greene and Cohen (2004, p. 1775) argue,

……neuroscience will probably have a transformative effect on the law, despite the fact that existing legal doctrine can, in principle, accommodate whatever neuroscience will tell us. New neuroscience will change the law, not by undermining its current assumptions, but by transforming people’s moral intuitions about free will and responsibility. This change in moral outlook will result not from the discovery of crucial new facts or clever new arguments, but from a new appreciation of old arguments, bolstered by vivid new illustrations provided by cognitive neuroscience.

In the same way, psychology can accommodate “whatever neuroscience will tell us” but it affects so many aspects of what it is to be human that we will undoubtedly shift our conceptions of ourselves in the process. And it may be just around those moral intuitions that we will be most likely to shift.

Hustvedt (2010), in seeking an answer to her strange episode of shaking, scoured multiple disciplines and medical practices for an account of her aﬄiction. The fact that no single one could provide her with a satisfactory account indicates just how, without rejecting a notion of something like a brain disease, it is a hopelessly incomplete explanation. It appeared to her, after the fact, as more of a “conversion disorder,” but this too was unsatisfactory. And so the brain sciences, as they reshape how we view, manipulate, understand and investigate brains will also reshape our explanatory categories, but in ways we are unlikely to foresee. Hustvedt’s (2010) account is so compelling because we can see the incomplete nature of the neurosciences just as that science grapples with a condition like the one Hustvedt (2010) described. And she recognizes that the condition is neither solely organic nor conscious/unconscious. It is both and neither, and we are in transition.

## Conflict of Interest Statement

The author declares that the research was conducted in the absence of any commercial or financial relationships that could be construed as a potential conflict of interest.

## Footnotes

1 The phrase “modern synthesis” is of course a reference to a term coined by Huxley (1942) who along with numerous others combined genetics with population biology to create the conditions for this synthesis. I am using it here in this sense, the unification of disparate elements into a single unified framework or theory.

2 It was Koch (1964) who noted in a rather wistful manner that psychology seemed entirely untouched by the debate unleashed by Snow’s (1959) thesis of the divide between the humanities and the sciences.

3 It should be noted that there are very straightforward claims to the contrary, namely, in favor of something like explanatory pluralism of the sort expressed by Dale et al. (2009).

4 I am grateful to a reviewer who noted that different memory systems have been proposed for very different purposes. These systems do not always compare easily and hence lack an overarching framework.

5 This is a hypothetical example.
